# A bitter pill to swallow? Impact of affective temperaments on treatment adherence: a systematic review and meta-analysis

**DOI:** 10.1038/s41398-022-02129-z

**Published:** 2022-09-02

**Authors:** Georgina Szabo, Michele Fornaro, Peter Dome, Szabolcs Varbiro, Xenia Gonda

**Affiliations:** 1 Doctoral School of Mental Health Sciences, Semmelweis University, Budapest, Hungary; 2 Department of Psychiatry, Federico II University of Naples, Naples, Italy; 3 Department of Psychiatry and Psychotherapy, Semmelweis University, Budapest, Hungary; 4 National Institute of Mental Health, Neurology and Neurosurgery, Budapest, Hungary; 5 Department of Obstetrics and Gynecology, Semmelweis University, Budapest, Hungary; 6 NAP-2-SE New Antidepressant Target Research Group, Hungarian Brain Research Program, Semmelweis University, Budapest, Hungary

**Keywords:** Depression, Human behaviour, Bipolar disorder

## Abstract

**Background:**

Predominant affective temperament may affect adherence to prescribed pharmacotherapeutic interventions, warranting systematic review and meta-analysis.

**Methods:**

The Scopus, Web of Science, PubMed, and OVID MedLine databases were inquired since inception up to 31st of March 2022 for records of any study design documenting quantitative evidence about affective temperaments as measured by the Temperament Evaluation of Memphis, Pisa, Paris, and San Diego (TEMPS-A) questionnaire and treatment adherence measured by the means of major rating scales on the matter. People with low vs. high levels of treatment adherence, matched for otherwise clinically relevant variables, were deemed as cases and controls, respectively, using standardized mean differences (SMDs) in pertinent scores under random-effects meta-analysis.

**Results:**

Nine studies encompassing 1138 subjects pointed towards significantly higher cyclothymic (SMD = −0.872; CI: [−1.51 to −0.24]; *p* = 0.007), irritable (SMD = −0.773; CI: [−1.17 to −0.37]; *p* < 0.001) and depressive (SMD = −0.758; CI: [−1.38 to −0.14]; *p* = 0.017) TEMPS-A scores both for psychiatric and nonpsychiatric samples with poorer adherence.

**Limitations:**

Intrinsic limitations of the present report include the heterogeneity of the operational definitions documented across different primary studies, which nonetheless reported on the sole medication-treatment adherence, thus limiting the generalizability of the present findings based on a handful of comparisons.

**Conclusions:**

Though further primary studies need to systematically account for different clinical and psychosocial moderators across different clinical populations and operational definitions, cyclothymic, depressive, and irritable temperament scores may nonetheless predict treatment adherence and, thus, overall treatment outcomes.

## Introduction

Affective temperaments (namely depressive, cyclothymic, hyperthymic, irritable, and anxious) represent relatively stable [1] —though stressor-sensitive [2]—biological “cores” of personality developing early during the lifespan, accounting for much of the individual activity level, rhythms, moods, and related cognitions according to their classical Akiskalian conceptualization rooting back to Greek psychological medicine and philosophy [3].

Though void of any pathological value per se, affective temperaments may nonetheless represent vulnerability factors towards the development of various configurations of mood [4] as well as other psychiatric disorders [5] and specific somatic conditions [6–17]. Affective temperaments may also affect the long-term course and treatment outcome of various conditions, although evidence modeling the relationship between affective temperaments and the prognosis of somatic diseases is tentative [13, 15, 18, 19].

The role of affective temperaments in prognosis and illness course in somatic illnesses in part may be related to their impact on complying with treatment recommendations. Adherence is the degree to which a patient follows therapeutic advice, including recommended lifestyle changes such as diet or exercise beyond the prescribed drugs [20]. According to the World Health Organization (WHO) statistics, the rate of adherence to long-term treatment of chronic diseases in developed countries does not exceed 50%, and in developing countries, the rate is even lower. Inadequate adherence to long-term therapies seriously jeopardizes the effectiveness of treatment and is, therefore, a critical issue in the health of the population for both qualities of life and health economics fields [20].

Affective temperaments can be readily assessed by broadly validated tools such as the Temperament Evaluation of Memphis, Pisa, Paris, and San Diego Autoquestionnaire (TEMPS-A) [21, 22], with recent evidence suggesting their influence on treatment adherence [23–32]. The identification of high-risk subgroups with critical treatment adherence should, theoretically, inform the treatment plan, ideally aiding the personalized medicine approach and enhancing the cost-effective interventions.

Despite the associated clinical and public health burden, most of the available studies documenting treatment adherence in relationship with affective temperaments is hampered by low statistical power, essentially due to the small representativeness of the samples, or they rely on inhomogeneous ratings for the same outcomes.

Both of the latter issues could nonetheless be effectively addressed using a meta-analytic approach, providing a meaningful overall effect estimation from pooling low statistical original studies applying different versions of the TEMPS using SMD (standardized mean difference). The aim of the present study was to investigate the effect of affective temperaments on medication adherence in psychiatric as well as in nonpsychiatric patients using a systematic review and meta-analysis approach.

## Methods

The present systematic review and meta-analysis follow the Preferred Reporting Items for Systematic Reviews and Meta-Analyses (PRISMA), 2020 edition [33]. The review was not pre-registered.

### Search strategy

We searched the Scopus, Web of Science, PubMed, and OVID MedLine databases from inception up to 31st of March 2022. The following search terms were used: “TEMPS-A” AND (“adherence” OR “compliance”) followed by manual search and cross-references validation.

### Study selection

After pooling all the publications identified by the literature search across different databases, duplicate records were removed using a reference management software (EndNote v.20, Clarivate Analytics) [34] before fine manual review for duplicates. Two authors (GS and PD) performed the title and abstract screening, extracting the relevant full-texts using a-priori built extraction form (see below), and also assessed study eligibility and extracted data from the selected studies independently. Any eventual discrepancy between the reviewing authors was solved by consensus by inquiring a third author with considerable expertise in the field (XG).

No study design, age group, treatment modality, or publication language was applied. Contact with the authors was planned as necessary.

As our search terms included “TEMPS-A”, no qualitative studies were identified. We included studies that provided quantitative data both for the specific affective temperament types (measured with the TEMPS-A questionnaire) and for treatment adherence or compliance (measured with any adherence scale). For analysis of the effect size, associations of TEMPS-A scores and treatment adherence (e.g., mean difference, correlation) were also required.

### Data extraction and analysis

From all included articles, we systematically extracted the following data: country of origin, characteristics of the patient populations (sample size, sex ratio, age, type of patient population), affective temperaments measurement scale, adherence measurement method and scale, and the reported mean affective temperament subscale ratings of the adherent and non-adherent patient groups with the corresponding indicator of standard deviation (SD), or the correlation coefficient of the different affective temperament subscale ratings and the adherence score. In those studies, where both mean and correlation was presented, we chose correlation to minimize inconsistency caused by the arbitrarily defined cut-points used to dichotomize adherence scales into adherent and non-adherent groups. In those studies, where more than two adherence subgroups were compared, or the means were reported separately by sex, we merged subgroups by calculating the weighted mean and pooled SD for the given related groups. For the meta-analysis, the extracted values were converted to standardized mean differences (SMDs) and standard deviations (SDs) for all included studies. Since we assumed significant methodological heterogeneity due to the lack of a uniform adherence measurement method, we used the random-effects method for meta-analytic pooling, based on SMDs in scores between adherent and non-adherent subjects. Meta-analytic findings are reported as pooled-SMDs with 95% confidence intervals (CIs).

Between-study heterogeneity was determined by calculating the prediction interval of the distribution of true effects. The I^2^, Q, and Tau^2^ statistics were likewise computed (where the Q-statistic provides a test of the null hypothesis that all studies in the analysis share a common effect size; I^2^ tells us what percentage of the variance in observed effects reflects variance in true effects rather than sampling error, and Tau^2^ is the variance of the true effects sizes) [35]. Possible causes of heterogeneity were explored by visual inspection of forest plots looking for outlier values and by subgroup analysis. Also, possible moderators were investigated by meta-regression analysis. Patient population, age, sex, and country of origin were analysed as possible moderators for all temperament subscales separately.

Quality and risk of bias within studies was assessed using the NIH Quality Assessment Tool for Observational Cohort and Cross-Sectional Studies [36]. Publication bias were assessed using funnel plot techniques, Begg and Mazumdar’s method, and Egger’s regression test. Sensitivity analysis was performed to test the impact of the individual studies and also the impact of the effect size index selection.

All statistical analyses were implemented using the R package metaphor v.3.0 [37].

## Results

The adopted search strategy returned a total of 219 hits, resulting in 169 records after duplicate removal, which were screened on the title and abstract for inclusion criteria, out of which 147 were excluded for not providing required information. Twenty-two records were ultimately selected for detailed review and potential inclusion. In one of the identified studies, necessary data were only partially reported, but successful contact with the authors allowed for the inclusion of that record [24].

**Fig. 1:**
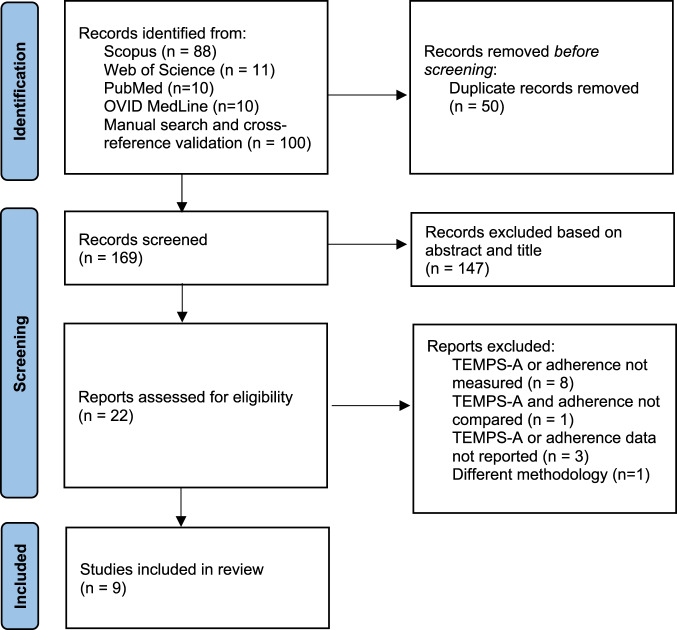
PRISMA flowchart. Flowchart of study identification and selection process.

Ten publications were found eligible for meta-analytic synthesis [23**–**26, 28–32, 38], though one record was ultimately disregarded since its adherence definition (treatment drop-out) significantly differed from the rest of the other included studies (medication adherence). Finally, nine records encompassing 1138 subjects effectively participated in the meta-analytic synthesis of the evidence (Fig. 1 and Table 1).

**Table 1:** Characteristics of the included studies.

Study	Subjects	Country	Age (mean)	Sex (f%)	Population	Affetive temperaments [scale]	Adherence [scale]	Statistics used	Adh-AT related findings	Study quality
Belvederi Murri et al., 2017	279	Italy	57.45	48.02	Nonpsychiatric (Diabetes type 1 and 2)	TEMPS-A-39 [*Z*-score]	MMAS-4 [0–4] high score: low adh	correlation	irr - low adh dep - low adh cyc - low adh	I
Bahrini et al., 2015	36	Tunisia	37	33.3	Psychiatric (various)	TEMPS-A (Lebanese) [0, 25]	MARS (medication adh scale) [1–4] high score: high adh	correlation	irr - low adh dep - low adh	II
Kamei et al., 2013	38	Japan	52.2	39.47	Psychiatric (various)	TEMPS-A [1, 2]	VAS (medication adh scale) [0–100] high score: high adherence	correlation	irr - low adh	II
Fornaro et al., 2013	220	Italy	38.95	58.63	Psychiatric (Bipolar Depression type II)	TEMPS-A (Rome) [0, 25]	MMAS-8 + CRS (combined) [0–8] high score: high adherence	mean difference (cut point: 5)	cyc - low adh irr - low adh dep - low adh anx - low adh hyp - low adh	I
Shamsi et al., 2014	207	Iran	48.4	61.8	Nonpsychiatric (Diabetes type 2)	TEMPS-A [1, 2]	Likert scale [1–5] high score: high adherence	mean difference (cut point: 4)	cyc - low adh irr - low adh dep - low adh anx - low adh	I
Shamsi et al., 2021	150	Iran	48.48	54.66	Nonpsychiatric (Congestive Heart Failure)	TEMPS-A-35 [0, 1]	MMAS-8 [0–8] high score: high adherence	mean difference (cut point: 6)	irr - low adh	I
Yamamoto et al., 2021	54	Japan	58.94	43.89	Nonpsychiatric (Diabetes type 2)	TEMPS-A [*Z*-score]	MMAS-4 [0–4] high score: low adherence	correlation mean difference (cut point: 2)	cyc - low adh	I
Buturak et al., 2016	80	Turkey	40.74	57.5	Psychiatric (Bipolar Depression type I)	TEMPS-A (Turkish) [0, 25]	MMAS-4 [0–4] high score: low adherence	correlation mean difference (cut point: 1)	cyc - low adh irr - low adh dep - low adh anx - low adh	II
Pasquale et al., 2016	74	Italy	48.3	42.25	Nonpsychiatric (Kidney transplant)	TEMPS-A [0, 1]	BAASIS (“taking” scale) [0,5] high score: low adherence	correlation	cyc - low adh irr - low adh dep - low adh	I
**Totals (k** **=** **9)**	**1138**	**Five countries**			**Psychiatric (4)** **Nonpsychiatric (5)**	**TEMPS-A (7)** **TEMPS-A short (2)**	**Various scales, all addressing medication adh**	**Correlation (4)** **Mean difference (3)** **Both (2)**	**8/9 (89%) irr - low adh** **6/9 (67%) dep - low adh** **3/9 (33%) anx - low adh** **1/9 (11%) hyp - low adh**	

*TEMPS-A* Temperament Evaluation of Memphis, Pisa, Paris, and San Diego–Autoquestionnaire version, *MMAS* Morisky MedicationAdherence Scale, *MARS* Medication Adherence Rating Scale, *CRS* Clinician Rating Scale, an ordinal scale of 1–7 to quantify the clinician’s assessment of the level of adherence, *BAASIS* Basel Assessment of Adherence to Immunosuppressive Medication Instrument, *cyc* cyclothymic, *irr* irritable, *dep* depressive, *anx* anxious, *hyp* hyperthymic, *adh* adherence, *AT* affective temperaments, Study quality II: good, I: potential risks identified, 0:poor, based on JBI Critical Appraisal Checklist for Analytical Cross-Sectional Studies

Core characteristics of the nine analyzed reports [23**–**26, 28–32] appear in Table 1. All studies found significant associations between adherence and TEMPS-A scores for one or more of cyclothymic, depressive, anxious, irritable, or hyperthymic temperament across four psychiatric and five nonpsychiatric samples. TEMPS-A subscale scores were higher among non-adherent versus adherent subjects as follows: irritable (k = 8 out of 9 = 89%), cyclothymic (k = 6 out of 9 = 67%), depressive (k = 6 out of 9 = 67%), anxious (k = 3 out of 9 = 33%), hyperthymic (k = 1 out of 9 = 11%).

### Quality and risk of bias within studies

The risk of bias within studies was assessed by using the NIH Quality Assessment Tool for Observational Cohort and Cross-Sectional Studies [36] (Supplementary Table 1). According to our assessment, we identified potential risks of bias in seven studies [23**–**26, 28, 29, 31] due to using non-validated scales, applying the non-standardized definition of cut-points used to dichotomize adherence scales into adherent and non-adherent groups, or potential imprecision in reported data.

### Meta-analysis

Quantitative analysis was performed for each affective temperament type to test for differences in TEMPS-A subscale scores between adherent and non-adherent subjects. A negative effect represents that subjects with higher TEMPS-A scores favoring lower adherence. All results are reported in detail in Table 2, and those of particular interest are summarized below.

**Table 2:** Summary of meta-analyses of TEMPS-A ratings associated with adherence*.

Temperament	Sample size (*n*)		Effect size (pooled SMD)		Test of null		Heterogeneity			True effect size	
	Studies	Subjects	SMD	(95% CI)	Z	p(Z)	Q	p(Q)	I2	Tau2	(95% PI)
Cyclothymic	9	1138	**−0.869**	(−1.54 to −0.2)	−2.54	**0.011**	157.21	**0.000**	**95.52**	0.99	(−2.93 to 1.19)
Irritable	9	1138	**−0.772**	(−1.14 to −0.4)	−4.11	**0.000**	64.20	**0.000**	**85.65**	0.26	(−1.84 to 0.29)
Depressive	9	1138	**−0.756**	(−1.39 to −0.12)	−2.34	**0.019**	154.19	**0.000**	**95.14**	0.88	(−2.7 to 1.19)
Anxious	9	1138	−0.248	(−0.52 to 0.02)	−1.80	0.072	29.47	0.000	74.38	0.12	(−0.97 to 0.48)
Hyperthymic	9	1138	0.045	(−0.34 to 0.43)	0.23	0.817	44.21	0.000	87.18	0.28	(−1.06 to 1.15)

^*^Based on standardized mean differences (SMD) in TEMPS-A scores in random-effects meta-analysis. *CI* confidence interval, tells us how precisely we have estimated the mean effect, *Z-statistic* test of the null hypothesis that effect size is zero, rejected if *p* < 0.05; *Q-statistic* test of the null hypothesis that all studies in the analysis share a common effect size, rejected if *p* < 0.05; *I*^*2*^ percentage of the variance in observed effects reflects variance in true effects rather than sampling error, *Tau*^*2*^ the variance of the true effect sizes, *PI* prediction interval, tells us how the true effect size varies across populations.

Significant effects (*p* < 0.05) and heterogeneity are marked in bold.

Based on nine studies meeting inclusion criteria (total *n* = 1138 subjects), patients with lower adherence had significantly higher cyclothymic (SMD = −0.869, CI: [−1.54 to −0.2], *p* = 0.011), irritable (SMD = −0.772 [CI: −1.14 to −0.4], *p* < 0.001) and depressive (SMD = −0.756, CI: [−1.39 to −0.12], *p* = 0.019) TEMPS-A scores compared to adherent subjects. Anxious (*p* = 0.072) and hyperthymic (*p* = 0.817) TEMPS scores were not different between the two groups.

Heterogeneity was high for all temperaments subscales (I^2^ = 88–95%, *p* < 0.001), with wide prediction intervals for the true effect size estimation, including zero, suggesting that true effects vary from around −2.9 SMD in some populations to 1.2 SMD in others.

**Fig. 2:**
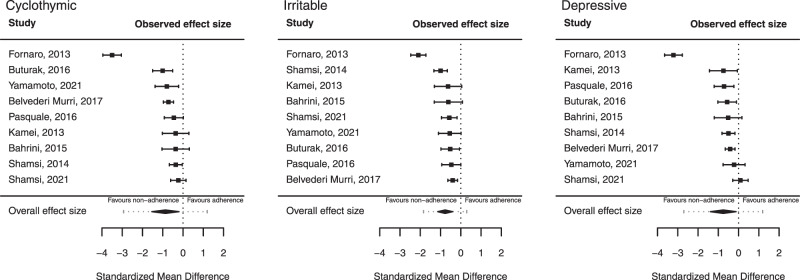
Meta-analysis of studies investigating the effect of affective temperaments on medication adherence. Forest plots based on random-effects meta-analyses of TEMPS-A scores for cyclothymic, irritable, and depressive temperaments with 95% confidence intervals (CIs) in nine comparisons of adherent versus non-adherent subjects (total *n* = 1138), with pooled standardized mean differences (SMDs). The estimated 95% prediction interval (PI) likewise presented, in which the true effect size was predicted to fall in 95% of all comparable populations.

Forest plots with pooled SMDs, 95% CIs, and 95% prediction intervals (PIs) for the relevant temperament subscales (where statistically significant associations of affective temperament scores with adherence were found) are presented in Fig. 2.

### Sensitivity analysis

#### Impact of one outlying study

The inspection of the forest plots (Fig. 2) revealed one outlying study [29], which reported a much bigger effect size than the rest of the others. Meta-analysis was repeated with the exclusion of the latter study. The main findings of the meta-analysis without the one outlier study are summarized in Table 3.

**Table 3:** Summary of meta-analyses of TEMPS-A ratings associated with adherence (one outlier removed)*.

Temperament	Sample size (*n*)		Effect size (pooled SMD)		Test of null		Heterogeneity			True effect size	
	Studies	Subjects	SMD	(95% CI)	Z	p(Z)	Q	p(Q)	I2	Tau2	(95% PI)
Cyclothymic	8	918	**−0.538**	(−0.73 to −0.35)	−5.48	**0.000**	10.59	0.157	36.61	0.03	(−0.91 to −0.17)
Irritable	8	918	**−0.591**	(−0.78 to −0.41)	−6.29	**0.000**	8.60	0.283	32.45	0.02	(−0.93 to −0.25)
Depressive	8	918	**−0.414**	(−0.61 to −0.22)	−4.25	**0.000**	10.52	0.161	37.25	0.03	(−0.79 to −0.04)
Anxious	8	918	−0.192	(−0.48 to 0.09)	−1.31	0.189	23.07	0.002	72.25	0.11	(−0.92 to 0.53)
Hyperthymic	8	918	0.042	(−0.1 to 0.18)	0.59	0.557	17.59	0.014	0.00	0.00	(−0.1 to 0.18)

^*^Based on standardized mean differences (SMD) in TEMPS-A scores in random-effects meta-analysis. *CI* confidence interval, tells us how precisely we have estimated the mean effect*, Z-statistic* test of the null hypothesis that effect size is zero, rejected if *p* < 0.05, *Q-statistic* test of the null hypothesis that all studies in the analysis share a common effect size, rejected if *p* < 0.05, *I*^*2*^ percentage of the variance in observed effects reflects variance in true effects rather than sampling error, *Tau*^*2*^ the variance of the true effect sizes, *PI* prediction interval, tells us how the true effect size varies across populations.

Significant effects (*p* < 0.05) and heterogeneity are marked in bold.

Exclusion of the outlying study from the meta-analysis resulted in a still large sample (k = 8, *n* = 918), insignificant heterogeneity (I^2^ = 32–37%, *p* > .05), and a somewhat reduced but still significant negative meta-analytic association for cyclothymic, irritable and depressive temperaments. Estimated PIs do not include zero anymore, suggesting that in 95% of all comparable populations, a negative true effect exists.

**Fig. 3:**
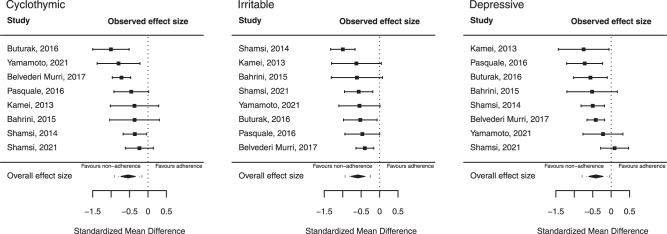
Meta-analysis of studies investigating the effect of affective temperaments on medication adherence (one outlier removed). Forest plots without the one excluded study, based on random-effects meta-analyses of TEMPSA scores for cyclothymic, irritable, and depressive temperaments with 95% CIs in eight comparisons of adherent versus non-adherent subjects (total *n* = 918), with pooled SMDs. The estimated 95% PIs also displayed, in which the true effect size was predicted to fall in 95% of all comparable populations.

Forest plots without the one outlier study with pooled SMDs, 95% CIs, and 95% prediction intervals for the relevant temperament subscales are presented in Fig. 3.

#### Impact of individual studies

In order to investigate the impact of each individual study on the summary estimate, sensitivity analysis was performed by iteratively excluding one study from the analyses and recalculating the overall SMDs. We performed analyses both with and without the excluded outlying study in order to make sure that its removal didn’t cause any bias (Supplementary Tables 2, 3). The overall SMDs did not vary substantially after excluding any individual study, indicating that the results were not driven by one of the analyzed individual studies, either with or without the excluded outlying study.

#### Impact of the selected effect size index

As presented in Table 1, the reported effect size indices were heterogeneous among the included studies, some measured effect size as SMD, while others as correlation. We decided to select SMD as our effect size index in the current analysis, and although correlation and SMD can be mathematically converted to each other, this conversion might have an impact on the results in some cases [39]. In order to investigate the impact of this selection on the summary estimates, sensitivity analysis was performed by recalculating all the reported results with correlation instead of SMD as the effect size index. We performed analyses both with and without the excluded outlying study in order to make sure that converting between effect size indices didn’t cause any bias (Supplementary Tables 4, 5). The overall results did not vary substantially with correlation as the effect size index, indicating that the results were not biased by the effect size selection.

### Publication bias

Publication bias analysis was performed on 8 studies after the removal of one outlier [23**–**26, 28, 30–32]. Based on inspection of funnel plots, and also on Egger’s regression and Begg and Mazumdar’s rank correlation tests (*p* = 0.976; 0.933; 0.497, and *p* = 1.000; 0.548; 0.398 for cyclothymic/irritable/depressive TEMPS-A subscales, respectively), there was no evidence of publication bias (Supplementary Fig. 1).

### Moderator variables

In case of cyclothymic, irritable, and depressive temperaments, meta-regression results didn’t show any moderating effect of age, sex, patient population, or country of origin. In case of anxious temperament subgroup analysis revealed that among psychiatric patients anxious temperament scores were significantly higher with medication non-adherence vs adherence (SMD = -0.593; CI: [−0.82 to −0.36]; *p* < 0.001), but not in nonpsychiatric population (SMD = −0.042; CI: [−0.38 to 0.3]; *p* = 0.807). In case of hyperthymic temperament, female sex moderated the effect on medication adherence (Slope = −0.0449, CI: [−0.08 to −0.01], *p* = 0.009, and Intercept = 2.269; CI: [0.55 to 3.98]; *p* = 0.009), *R*^2^ = 49.03%). All the meta-regression results are reported in Supplementary Table 6.

## Discussion

Based on nine studies meeting inclusion criteria involving 1138 subjects in total, strong, adverse associations of affective temperament scores with adherence were found for cyclothymic, irritable, and depressive temperaments both in psychiatric and nonpsychiatric patient samples either with or without the outlier study suggesting an involvement of these temperamental types in determining decreased adherence.

Therapeutic adherence, especially in various somatic and psychiatric disorders of a more chronic nature requiring longer-term or continuous maintenance therapy is pivotal to controlling the symptoms and preventing recurrences in the majority of disorders, influencing illness course and outcome, thus the success and safety of using the efficacious treatment and the long-term well-being of patients. Adherence to treatment is also strongly related to preventable healthcare costs related to relapses and recurrences, repeated hospital admissions, a chronic disease course, and resulting decreased productivity [23], amounting to an estimated 100 billion USD in the US alone [40].

Finding and selecting an efficacious pharmacological treatment is thus only one component of the therapeutic success, while the other, equally important and equally challenging, is the active participation of the patient in the form of adhering to medical recommendations. In case of all medical specialties combined, it is estimated that 30–50% of patients do not take their medications as prescribed [40]. Thus increasing the efficacy of adherence to interventions is an imminent public health challenge prompting the WHO to claim that improving adherence would have a significantly greater impact on the general health of the population than improvement in specific pharmacological and other medical treatments [20, 40].

The biopsychosocial model of illness acknowledges biological, psychological, and social aspects as equally important in understanding and explaining treatment adherence [41]. Therapy adherence is a highly complex process involving multiple contributing factors of both dynamic and static nature, including a complex pattern of factors related to the patient, the treatment, the doctor-patient relationship, and the environment, many of which are underlined by psychological characteristics of the patient. Moreover, as opposed to more concrete barriers to adherence, psychological barriers are more challenging and complex, thus also more difficult to identify and address [42]. Important components of these psychological barriers include or are determined by personality and temperamental factors of the patients, which have been less studied, even though these are strongly associated with adherence-related behaviors by determining emotions, cognitions, attitudes as well as reactions. It has increasingly been suggested that personality may have a significant impact on the long-term course and outcomes of several illnesses, and also medication adherence may, in fact, be an important factor mediating this effect [43]. Especially considering that the paradigm shift from compliance to adherence in describing the willingness of the patient to cooperate with the prescribed treatment was prompted by abandoning the conceptualization of the patient as the passive and obedient part in their treatment in favor of a more active role where the patient assumes a behavior matching the clinician's recommendation on all components of treatment including pharmacotherapy, lifestyle, as well as following up with appointments and further tests, we increasingly understand that good treatment management requires understanding of such patient characteristics as personal experience, disease-related beliefs, perception of health status, psychological state, as well as personality and temperamental factors of the patients [23].

Several models of medication adherence have been developed which aim to take the psychological characteristics of patients into consideration. These include for example the Health Belief Model by Rosenstock [44] highlighting the role of beliefs about susceptibility and seriousness of a health problem leading to a perception of threat which will be combined with perceived benefits and barriers of a course of action, a personal sense of self-efficacy, and environmental cues to action, together determining engagement in a behavior aimed at addressing the health threat; The Theory of Planned Behavior [45] which focuses on the role of intentions, shaped by attitudes, norms and perceived self-efficacy, in predicting adherence-related behaviors; the Necessity-Concerns Framework which suggests that beliefs about necessity of treatment are weighed against worries about adverse effects as the key determinants of decisions on adherence, or the Information-Motivation-Strategy model [46] which addresses cognitive, social and environmental factors postulating that the patient must have sufficient information on what they should do, have to possess the motivation to do it, and must have the strategy and means to actually execute it. The above models include several important aspects where temperamental and personality factors, and specifically affective temperaments, which determine emotional reactivity, related cognitions, and behaviors, may have a key contribution to actually determine adherence-related behaviors.

In light of the above, our results supporting that more marked irritable, cyclothymic and depressive affective temperaments are associated with decreased adherence may hold several important clinical and public health implications. The analysis and understanding of these factors and their impact on non-adherent behavior may provide important cues for both identifying the psychological support of patients to be able to better cooperate with therapy, as well as for psychological or psychiatric interventions if necessary to increase adherence. By identifying temperamental contributors for non-adherence, screening methods and risk indicators, as well as focused psychotherapeutic, education and supportive methods could also be developed. Thus, understanding the role of the core of personality, i.e., temperament and how they influence medication-related attitudes, beliefs, cognitions, emotions, and behaviors would provide a novel and crucial possibility to develop patient-tailored and personalized education, support, and intervention methods to target the obstacles of adhering to medical recommendations and increase the active participation of patients in their successful treatment.

Out of the five affective temperament types described by Akiskal, we saw the significant impact of three, namely, cyclothymic, irritable, and depressive, on an increased risk of non-adherence to therapy. Considering the characteristics of these affective temperamental types may provide some clues both on how they influence adherence and what types of interventions they allow for. In general, cyclothymic and irritable temperaments induce less favorable reactions toward disturbing events [30]. Corresponding to earlier definitions by Kraepelin, cyclothymic temperament is a constitutive predisposition toward intolerance of subjective pain as well as a tendency for enhanced emotional response upon intense stressful and painful experience [22, 29] and is also associated with hopelessness [47]. Thus, these temperaments would make it more difficult to tolerate the burden of taking long-term medications, accepting the unchangeable fact of illness and sustained or even lifelong adherence to the required medication regime, and make also the acceptance of having to tolerate side effects more difficult.

Nevertheless, before further interpreting the results, we must also mention the initially detected high heterogeneity and the possible underlying causes. Although in the current meta-analysis, heterogeneity turned out to be mostly driven by one outlying study, and its exclusion made the sample rather homogenous, the initial high heterogeneity was not unexpected due to the different patient populations, the various methods used to measure adherence, and the fact, that adherence in clinical practice is a combined and dimensional construct with several influencing factors.

The issue of various measurement methods is not specific to this particular study, according to a recent systematic review of the existing adherence measurement scales, there are at least 121 patient-reported outcome measures on medication adherence in clinical use with various levels of consistency of their different psychometric properties [48]. In our current analysis, all nine included studies used different methods for recording adherence. Six different scales were used (MMAS, MARS, BAASIS, VAS, CRS, Likert scale), also different versions of one scale and different cut-off points were used to dichotomize adherence data. But despite all of this variability of adherence measurement methods, our results suggest that, in fact, they all addressed the same phenomenon because, with the removal of only one outlier study, heterogeneity between the rest of the studies became insignificant.

Regarding different patient populations, high heterogeneity was also presumed. Although emerging evidence supports the influence of temperament on treatment adherence both in psychiatric and nonpsychiatric populations, there are several other influencing factors, some of which may be deeply related to the different kinds of diseases and illness phases. For instance, in psychiatric practice, adherence to antipsychotics is highly related to a substantial lack of insight [49], whereas poor adherence in depression may be more related to cognitive aspects rather than a lack of will to step out of acute depression [50]. Also, in case of psychotropic medications of any kind, fear of stigmatization can play a role in non-adherence [50], while this factor is not relevant in case of chronic somatic diseases [51]. In case of diabetes treatment, the prevalence of non-adherence to insulin is higher compared to prescribed oral antidiabetics due to the fear of injections and the embarrassment related to injecting in public [52], which is also a unique factor related to this certain disease and more specifically to this certain prescribed medication. These are just a few examples of how different factors may underlie non-adherence in different patient groups. Unfortunately, current evidence doesn’t allow for such subtler distinction due to the lack of data for quantitative meta-analysis, however, the performed subgroup analysis suggests that the effect of cyclothymic, irritable, and depressive temperaments on adherence are not significantly different between psychiatric and nonpsychiatric populations. A possible explanation for this could be either that affective temperaments have a direct effect on adherence which is independent of the patient population, which can be explained by the general characteristics of these temperament types discussed earlier, or that affective temperaments have a different kind of indirect moderating effect on the different kind of underlying factors finally all resulting in non-adherence. For instance, among psychiatric patients, cyclothymic temperament has been found to be associated with negative attitudes toward psychotropic medications and their negative side effects, which may result in decreased adherence [32], while among patients with diabetes, cyclothymic temperament type has been associated with inadequate eating habits, which may also indirectly affect adherence to medical recommendations and disease outcome [24]. Both assumptions (direct and indirect effects of affective temperaments) are presumably valid, but we still have quite little knowledge about the causal relations behind.

In fact, there should be several additional moderators and mediators which may moderate (or may be in turn moderated by) the biological effect modifiers (affective temperaments, in this instance) and, therefore, might also explain the dispersion of real effects and as such, the initially detected heterogeneity and the observed effect size of the outlier study. A number of patient-related risk and protective factors for adherence have been identified so far—we already mentioned some of them earlier—that can possibly be also influenced by affective temperaments to some extent. These include, but are not limited to lower socioeconomic status, ethnicity, the impact of local cultural norms, social pressure or stigmatization, adolescence and old age, loneliness in old age, psychiatric disorders associated with the disease, personality disorders, comorbid somatic diseases, drug use, cognitive impairment, pregnancy, disease severity, poor insight and negative drug-related beliefs [50]. Also, while affective temperaments should hold relatively stable during the lifespan, they have also been found to be sensitive to certain factors, such as age, sex, or severity of illness [53].

In the present study, moderator analysis hasn’t identified any moderators in case of cyclothymic, irritable, and depressive temperaments, while anxious predominant temperament was found to be a possible risk factor for treatment non-adherence in psychiatric populations and hyperthymic predominant temperament as a possible protective factor against non-adherence among male subjects. It is important to note that the number of eligible studies may not have provided enough statistical power to examine moderating factors by meta-regression, furthermore, in some of the original studies, adherence and affective temperaments were not the measures of interest, but they themselves were the confounding or moderator variables, therefore no data was available on additional factors possibly moderating the affective temperament-adherence relationship.

As we discussed above, adherence is a complex construct with several possible moderating and mediating factors, so research in the next step should focus on identifying these external factors indirectly affecting temperament expressions or factors moderating their effect on treatment adherence, also delineating the different causal processes behind the non-adherence—temperament relationship in case of different diseases. Especially because affective temperaments are strongly genetically- and biologically based, exhibit a stable course throughout the lifespan, and are relatively unmodifiable directly using psychological and psychotherapeutic technics, so even though they are somewhat sensitive to external stimuli and stressors, changing them cannot be a realistic goal even in case of a patient-tailored and personalized therapy. However, we also see that although temperaments may affect adherence directly to some extent through behavior consistent with those traits (e.g., patients with cyclothymic temperament might be more intolerant of subjective pain), temperament most often not directly, but indirectly affects adherence by shaping behavior through different perceptions and processing of environmental stimuli [54, 55], which mediating factors, in turn, can already be modified through psychological and psychotherapeutic techniques.

Thus, rather than affective temperaments being the focus of psychotherapy themselves, we should first identify these mediating and moderating factors followed by targeting those with educational, supportive, and psychotherapeutic techniques in order to improve treatment adherence and overall treatment outcomes as a consequence. We should also understand how the genetic background and socioeconomic contributors to the development of temperaments may be related to later medication adherence.

### Limitations

Intrinsic limitations of the present study essentially rely on the paucity of evidence eligible for inclusion as well as the various thresholds set by the authors to operationally defined adherence outcomes.

### Conclusions

To the best of our knowledge, this is the first meta-analysis to show that affective temperament scores measured by the TEMPS-A scale can contribute to identifying the risk of medication non-adherence. Though further primary studies need to systematically account for different clinical and psychosocial moderators across different clinical populations and operational definitions, cyclothymic, depressive, and irritable temperament scores may nonetheless predict treatment adherence and, thus, overall treatment outcomes. In clinical practice, the TEMPS-A scale might be useful for screening patients before treatment in order to identify non-adherent high-risk groups and support them to increase their adherence. The real goal for forthcoming ecological studies is to explain the variance of effect modifier, i.e. affective temperament beyond other confounding factors across different settings. While affective temperaments should hold relatively stable during the lifespan, they may nonetheless be sensitive to intense environmental stressors, thus understanding the residual variance of modifiable factors is crucial from a public health perspective.

## Supplementary information

SUPPLEMENTARY INFORMATION
