# Supplementary material for: A bitter pill to swallow? Impact of affective temperaments on treatment adherence: a systematic review and meta-analysis
Source: Transl Psychiatry. 2022 Sep 2;12:360. doi: 10.1038/s41398-022-02129-z (PMC9440110; doi:10.1038/s41398-022-02129-z)
Supplement: Supplementary file 1 — SUPPLEMENTARY INFORMATION [file 41398_2022_2129_MOESM1_ESM.docx]

# SUPPLEMENTARY INFORMATION

**A bitter pill to swallow? Impact of affective temperaments on treatment adherence: A Systematic Review and Meta-Analysis**

Georgina Szabo, Michele Fornaro, Peter Dome, Szabolcs Varbiro, Xenia Gonda

Table of contents:

[Supplementary Fig. 1. Funnel plot of the studies that were included in the comparison of (relevant) TEMPS-A subscale scores and adherence. (k=8, Egger’s test: p=0.976; 0.933; 0.497, Begg and Mazumdar’s test: p=1.000; 0.548; 0.398 for cyclothymic/ irritable/ depressive TEMPS-A subscales, respectively). 2](#_Toc106916840)

[Supplementary Table 1. Quality assessment of the studies included in the meta-analysis using the NIH Quality Assessment Tool for Observational Cohort and Cross-Sectional Studies*. 2](#_Toc111578097)

[Supplementary Table 2. Sensitivity analysis: one-study-excluded statistics for the TEMPS-A ratings associated with adherence (outlier included). 3](#_Toc111578098)

[Supplementary Table 3. Sensitivity analysis: one-study-excluded statistics for the TEMPS-A ratings associated with adherence (outlier excluded). 3](#_Toc111578099)

[Supplementary Table 4. Summary of meta-analyses of TEMPS-A ratings associated with adherence using correlation as effect size index*. 4](#_Toc111578100)

[Supplementary Table 5. Summary of meta-analyses of TEMPS-A ratings associated with adherence using correlation as effect size index (one outlier removed)*. 4](#_Toc111578101)

[Supplementary Table 6. Meta-regression of moderators of TEMPS-adherence association. 5](#_Toc111578102)

[Supplementary Table 7. 2020 PRISMA checklist. 5](#_Toc111578103)

Supplementary Table 1. Quality assessment of the studies included in the meta-analysis using the NIH Quality Assessment Tool for Observational Cohort and Cross-Sectional Studies*.

|  | Study design | Research question clearly stated | Study population clearly defined? | Participation rate at least 50% | Subjects selected from the same population | Sample size justification, power description provided | Exposure(s) measured prior to the outcome(s) | Timeframe sufficient | Different levels of the exposured examined | Exposure measures clearly defined, valid, reliable | Exposure(s) assessed more than once | Outcome measures clearly defined, valid, reliable | Outcome assessors blinded | Loss to follow-up after baseline 20% or less | Confounding variables measured, adjusted | | Overall quality |
| --- | --- | --- | --- | --- | --- | --- | --- | --- | --- | --- | --- | --- | --- | --- | --- | --- | --- |
| Belvederi Murri et al., 2017 | cross- sectional | yes | yes | yes | yes | yes | no | n/a | yes | Short version of TEMPS-A scale used | no | yes | no | n/a | yes | | I |
| Bahrini et al., 2015 | cross- sectional | yes | yes | n/r | yes | no | no | n/a | yes | yes | no | yes | no | n/a | no | | II |
| Kamei et al., 2013 | cross- sectional | yes | yes | n/r | yes | no | no | n/a | yes | yes | no | Subjective adherence measurement (VAS scale) | no | n/a | no | | I |
| Fornaro et al., 2013 | cross- sectional | yes | yes | yes | yes | no | no | n/a | yes | yes | no | Non-validated adherence scales used (2 different scales combined), adherence scale dichotomized | no | n/a | yes | | I |
| Shamsi et al., 2014 | cross- sectional | yes | yes | yes | yes | no | no | n/a | yes | yes | no | Non-validated adherence scale used (likert scale), Adherence scale splitted into 3 groups | no | n/a | yes | | I |
| Shamsi et al., 2021 | cross- sectional | yes | yes | yes | yes | no | no | n/a | yes | Short version of TEMPS-A scale used | no | Adherence scale dichotomized | no | n/a | yes | | I |
| Yamamoto et al., 2021 | case- control | yes | yes | no | yes | no | no | n/a | yes | Data of interest not reported, data provided by email upon request -> final data not peer-reviewed | no | Adherence scale not validated in Japanese language | no | n/a | yes | | I |
| Buturak et al., 2016 | cross- sectional | yes | yes | n/r | yes | no | no | n/a | yes | yes | no | yes | no | n/a | no | | II |
| Pasquale et al., 2016 | cross- sectional | yes | yes | n/r | yes | no | no | n/a | yes | Data available only on chart, may not be precise | no | Data available only on chart, may not be precise | no | n/a | yes | | I |
| *Overall quality reflects the circumstances of the assessment of the affective temperament-adherence association Overall quality: II: good, I: potential risks identified, 0:poor; n/a: not applicable, n/r: not reported | | | | | | | | | | | | | | | |  |  |

Supplementary Fig. 1. Funnel plot of the studies that were included in the comparison of (relevant) TEMPS-A subscale scores and adherence. (k=8, Egger’s test: p=0.976; 0.933; 0.497, Begg and Mazumdar’s test: p=1.000; 0.548; 0.398 for cyclothymic/ irritable/ depressive TEMPS-A subscales, respectively).

Supplementary Table 2. Sensitivity analysis: one-study-excluded statistics for the TEMPS-A ratings associated with adherence (outlier included).

| Study | Statistics with study removed (cyc) | | | | | Statistics with study removed (irr) | | | | | Statistics with study removed (dep) | | | | |  |
| --- | --- | --- | --- | --- | --- | --- | --- | --- | --- | --- | --- | --- | --- | --- | --- | --- |
|  | SMD | 95% CI | | Z | p | SMD | 95% CI | | Z | p | SMD | 95% CI | | Z | p |  |
| Kamei, 2013 | -0.929 | -1.67 | -0.18 | -2.45 | 0.014 | -0.786 | -1.19 | -0.38 | -3.79 | 0.000 | -0.757 | -1.47 | -0.04 | -2.08 | 0.037 |  |
| BelvederiM, 2017 | -0.888 | -1.65 | -0.13 | -2.28 | 0.022 | -0.826 | -1.23 | -0.42 | -4.00 | 0.000 | -0.801 | -1.52 | -0.09 | -2.20 | 0.028 |  |
| Fornaro, 2013 | -0.538 | -0.73 | -0.35 | -5.48 | 0.000 | -0.591 | -0.78 | -0.41 | -6.29 | 0.000 | -0.414 | -0.61 | -0.22 | -4.25 | 0.000 |  |
| Shamsi, 2021 | -0.951 | -1.69 | -0.21 | -2.52 | 0.012 | -0.798 | -1.21 | -0.38 | -3.78 | 0.000 | -0.865 | -1.54 | -0.19 | -2.50 | 0.012 |  |
| Shamsi, 2014 | -0.935 | -1.68 | -0.19 | -2.45 | 0.014 | -0.740 | -1.15 | -0.33 | -3.50 | 0.000 | -0.790 | -1.51 | -0.07 | -2.16 | 0.031 |  |
| Buturak, 2016 | -0.852 | -1.61 | -0.09 | -2.20 | 0.028 | -0.803 | -1.21 | -0.39 | -3.84 | 0.000 | -0.780 | -1.50 | -0.06 | -2.14 | 0.033 |  |
| Bahrini, 2015 | -0.929 | -1.67 | -0.19 | -2.45 | 0.014 | -0.787 | -1.19 | -0.38 | -3.81 | 0.000 | -0.784 | -1.49 | -0.07 | -2.17 | 0.030 |  |
| Pasquale, 2016 | -0.921 | -1.67 | -0.17 | -2.40 | 0.016 | -0.809 | -1.22 | -0.40 | -3.90 | 0.000 | -0.761 | -1.48 | -0.04 | -2.08 | 0.038 |  |
| Yamamoto, 2021 | -0.877 | -1.63 | -0.12 | -2.27 | 0.023 | -0.798 | -1.21 | -0.39 | -3.83 | 0.000 | -0.822 | -1.52 | -0.12 | -2.30 | 0.022 |  |
| The table contains the meta-analytic SMDs of TEMPS-A scores between adherent and non-adherent subjects with the exclusion of each study, its confidence intervals, Z-transformed values, and its significance level. SMD: standardized mean difference (point estimate); CI: confidence interval; Z-statistic: test of the null hypothesis that effect size is zero, rejected if p<0.05; cyc: cyclothymic; irr: irritable; dep: depressive | | | | | | | | | | | | | | | | |

Supplementary Table 3. Sensitivity analysis: one-study-excluded statistics for the TEMPS-A ratings associated with adherence (outlier excluded).

| Study | Statistics with study removed (cyc) | | | | | Statistics with study removed (irr) | | | | | | Statistics with study removed (dep) | | | | | |
| --- | --- | --- | --- | --- | --- | --- | --- | --- | --- | --- | --- | --- | --- | --- | --- | --- | --- |
|  | SMD | 95% CI | | Z | p | SMD | 95% CI | | Z | p | SMD | | 95% CI | | Z | p |  |
| Kamei, 2013 | -0.551 | -0.76 | -0.34 | -5.22 | 0.000 | -0.589 | -0.78 | -0.39 | -5.90 | 0.000 | -0.392 | | -0.59 | -0.19 | -3.86 | 0.000 |  |
| BelvederiM, 2017 | -0.484 | -0.70 | -0.27 | -4.46 | 0.000 | -0.658 | -0.86 | -0.46 | -6.49 | 0.000 | -0.420 | | -0.66 | -0.18 | -3.37 | 0.001 |  |
| Shamsi, 2021 | -0.594 | -0.79 | -0.40 | -6.09 | 0.000 | -0.597 | -0.81 | -0.38 | -5.52 | 0.000 | -0.486 | | -0.64 | -0.33 | -6.29 | 0.000 |  |
| Shamsi, 2014 | -0.578 | -0.79 | -0.36 | -5.23 | 0.000 | -0.484 | -0.64 | -0.33 | -6.07 | 0.000 | -0.401 | | -0.63 | -0.17 | -3.41 | 0.001 |  |
| Buturak, 2016 | -0.487 | -0.67 | -0.30 | -5.09 | 0.000 | -0.601 | -0.81 | -0.40 | -5.73 | 0.000 | -0.397 | | -0.61 | -0.18 | -3.61 | 0.000 |  |
| Bahrini, 2015 | -0.551 | -0.76 | -0.34 | -5.24 | 0.000 | -0.590 | -0.79 | -0.40 | -5.93 | 0.000 | -0.409 | | -0.62 | -0.20 | -3.88 | 0.000 |  |
| Pasquale, 2016 | -0.549 | -0.77 | -0.33 | -4.93 | 0.000 | -0.608 | -0.81 | -0.40 | -5.86 | 0.000 | -0.377 | | -0.57 | -0.18 | -3.74 | 0.000 |  |
| Yamamoto, 2021 | -0.514 | -0.72 | -0.31 | -4.88 | 0.000 | -0.596 | -0.80 | -0.40 | -5.84 | 0.000 | -0.437 | | -0.65 | -0.23 | -4.04 | 0.000 |  |
| The table contains the meta-analytic SMDs of TEMPS-A scores between adherent and non-adherent subjects with the exclusion of each study, its confidence intervals, Z-transformed values, and its significance level. SMD: standardized mean difference (point estimate); CI: confidence interval; Z-statistic: test of the null hypothesis that effect size is zero, rejected if p<0.05; cyc: cyclothymic; irr: irritable; dep: depressive | | | | | | | | | | | | | | | | | |

Supplementary Table 4. Summary of meta-analyses of TEMPS-A ratings associated with adherence using correlation as effect size index*.

| Temperament | Sample size (n) | | Effect size (correlation) | | Test of null | | Heterogeneity | | | True effect size | | |
| --- | --- | --- | --- | --- | --- | --- | --- | --- | --- | --- | --- | --- |
|  | Studies | Subjects | z | (95% CI) | Z | p(Z) | Q | p(Q) | I2 | Tau2 | (95% PI) |  |
| Cyclothymic | 9 | 1138 | **-0.367** | ( -0.59 to -0.14 ) | -3.17 | **0.002** | 159.85 | **0.000** | **92.61** | 0.11 | ( -1.05 to 0.32 ) |  |
| Irritable | 9 | 1138 | **-0.345** | ( -0.48 to -0.21 ) | -4.86 | **0.000** | 52.09 | **0.000** | **79.41** | 0.03 | ( -0.73 to 0.04 ) |  |
| Depressive | 9 | 1138 | **-0.320** | ( -0.54 to -0.1 ) | -2.81 | **0.005** | 157.41 | **0.000** | **92.30** | 0.10 | ( -0.99 to 0.35 ) |  |
| Anxious | 9 | 1138 | -0.112 | ( -0.24 to 0.01 ) | -1.75 | 0.081 | 28.57 | 0.000 | 74.71 | 0.03 | ( -0.45 to 0.22 ) |  |
| Hyperthymic | 9 | 1138 | 0.034 | ( -0.15 to 0.22 ) | 0.37 | 0.710 | 48.68 | 0.000 | 88.19 | 0.06 | ( -0.49 to 0.56 ) |  |
| *Based on Fisher's correlation (z) between TEMPS-A and adherence scores in random-effects meta-analysis. CI: confidence interval, tells us how precisely we have estimated the mean effect; Z-statistic: test of the null hypothesis that effect size is zero, rejected if p<0.05; Q-statistic: test of the null hypothesis that all studies in the analysis share a common effect size, rejected if p<0.05; I2: percentage of the variance in observed effects reflects variance in true effects rather than sampling error; Tau2: the variance of the true effects sizes; PI:prediction interval, tells us how the true effect size varies across populations | | | | | | | | | | | | |

Supplementary Table 5. Summary of meta-analyses of TEMPS-A ratings associated with adherence using correlation as effect size index (one outlier removed)*.

| Temperament | Sample size (n) | | Effect size (correlation) | | Test of null | | Heterogeneity | | | True effect size | | |
| --- | --- | --- | --- | --- | --- | --- | --- | --- | --- | --- | --- | --- |
|  | Studies | Subjects | z | (95% CI) | Z | p(Z) | Q | p(Q) | I2 | Tau2 | (95% PI) |  |
| Cyclothymic | 8 | 918 | **-0.256** | ( -0.36 to -0.15 ) | -4.95 | **0.000** | 13.70 | 0.057 | 50.16 | 0.01 | ( -0.47 to -0.04 ) |  |
| Irritable | 8 | 918 | **-0.276** | ( -0.35 to -0.2 ) | -6.91 | **0.000** | 6.16 | 0.522 | 21.72 | 0.00 | ( -0.4 to -0.15 ) |  |
| Depressive | 8 | 918 | **-0.197** | ( -0.29 to -0.1 ) | -4.00 | **0.000** | 11.90 | 0.104 | 45.32 | 0.01 | ( -0.4 to 0 ) |  |
| Anxious | 8 | 918 | -0.090 | ( -0.23 to 0.05 ) | -1.28 | 0.201 | 23.28 | 0.002 | 73.30 | 0.03 | ( -0.44 to 0.26 ) |  |
| Hyperthymic | 8 | 918 | 0.082 | ( -0.08 to 0.24 ) | 1.02 | 0.308 | 21.89 | 0.003 | 79.94 | 0.04 | ( -0.33 to 0.5 ) |  |
| *Based on Fisher's correlation (z) between TEMPS-A and adherence scores in random-effects meta-analysis. CI: confidence interval, tells us how precisely we have estimated the mean effect; Z-statistic: test of the null hypothesis that effect size is zero, rejected if p<0.05; Q-statistic: test of the null hypothesis that all studies in the analysis share a common effect size, rejected if p<0.05; I2: percentage of the variance in observed effects reflects variance in true effects rather than sampling error; Tau2: the variance of the true effects sizes; PI:prediction interval, tells us how the true effect size varies across populations | | | | | | | | | | | | |

Supplementary Table 6. Meta-regression of moderators of TEMPS-adherence association.

| Moderator | B | 95% CI | | p | R^2^ |
| --- | --- | --- | --- | --- | --- |
| **Cyclothymic** |  |  |  |  |  |
| Age | 0.052 | -0.039 | 0.142 | 0.263 | 4.00% |
| Population | -0.828 | -2.128 | 0.471 | 0.211 | 8.20% |
| Sex | -0.042 | -0.113 | 0.03 | 0.257 | 3.20% |
| HDI | -4.892 | -15.556 | 5.773 | 0.369 | 0.00% |
| **Irritable** |  |  |  |  |  |
| Age | 0.036 | -0.011 | 0.082 | 0.131 | 20.00% |
| Population | -0.423 | -1.145 | 0.3 | 0.252 | 8.00% |
| Sex | -0.028 | -0.066 | 0.011 | 0.156 | 15.00% |
| HDI | -0.736 | -6.978 | 5.505 | 0.817 | 0.00% |
| **Depressive** |  |  |  |  |  |
| Age | 0.059 | -0.022 | 0.141 | 0.152 | 13.20% |
| Population | -0.937 | -2.107 | 0.233 | 0.116 | 17.50% |
| Sex | -0.027 | -0.098 | 0.043 | 0.446 | 0.00% |
| HDI | -4.373 | -14.523 | 5.777 | 0.398 | 0.00% |
| **Anxious** |  |  |  |  |  |
| Age | 0.024 | -0.011 | 0.059 | 0.178 | 18.00% |
| Population | **-0.518** | **-0.964** | **-0.071** | **0.023** | **48.80%** |
| Sex | -0.02 | -0.048 | 0.007 | 0.151 | 25.60% |
| HDI | 1.318 | -3.277 | 5.914 | 0.574 | 0.00% |
| **Hyperthymic** |  |  |  |  |  |
| Age | -0.011 | -0.069 | 0.048 | 0.723 | 0.00% |
| Population | 0.22 | -0.627 | 1.068 | 0.61 | 0.00% |
| Sex | **-0.045** | **-0.078** | **-0.011** | **0.009** | **49.00%** |
| HDI | -4.21 | -10.336 | 1.917 | 0.178 | 4.10% |
| HDi: Human Development Index | | | | | |

Supplementary Table 7. 2020 PRISMA checklist.

| Section and Topic | Item # | Checklist item | Location where item is reported |
| --- | --- | --- | --- |
| TITLE | | |  |
| Title | 1 | Identify the report as a systematic review. | Title |
| ABSTRACT | | |  |
| Abstract | 2 | See the PRISMA 2020 for Abstracts checklist. | Abstract |
| INTRODUCTION | | |  |
| Rationale | 3 | Describe the rationale for the review in the context of existing knowledge. | Introduction |
| Objectives | 4 | Provide an explicit statement of the objective(s) or question(s) the review addresses. | Introduction |
| METHODS | | |  |
| Eligibility criteria | 5 | Specify the inclusion and exclusion criteria for the review and how studies were grouped for the syntheses. | Methods: Study selection |
| Information sources | 6 | Specify all databases, registers, websites, organisations, reference lists and other sources searched or consulted to identify studies. Specify the date when each source was last searched or consulted. | Methods: Search strategy |
| Search strategy | 7 | Present the full search strategies for all databases, registers and websites, including any filters and limits used. | Methods: Search strategy |
| Selection process | 8 | Specify the methods used to decide whether a study met the inclusion criteria of the review, including how many reviewers screened each record and each report retrieved, whether they worked independently, and if applicable, details of automation tools used in the process. | Methods: Study selection, Data extraction and analysis |
| Data collection process | 9 | Specify the methods used to collect data from reports, including how many reviewers collected data from each report, whether they worked independently, any processes for obtaining or confirming data from study investigators, and if applicable, details of automation tools used in the process. | Methods: Study selection, Data extraction and analysis |
| Data items | 10a | List and define all outcomes for which data were sought. Specify whether all results that were compatible with each outcome domain in each study were sought (e.g. for all measures, time points, analyses), and if not, the methods used to decide which results to collect. | Methods: Data extraction and analysis |
|  | 10b | List and define all other variables for which data were sought (e.g. participant and intervention characteristics, funding sources). Describe any assumptions made about any missing or unclear information. | Methods: Data extraction and analysis |
| Study risk of bias assessment | 11 | Specify the methods used to assess risk of bias in the included studies, including details of the tool(s) used, how many reviewers assessed each study and whether they worked independently, and if applicable, details of automation tools used in the process. | Methods: Study selection, Data extraction and analysis |
| Effect measures | 12 | Specify for each outcome the effect measure(s) (e.g. risk ratio, mean difference) used in the synthesis or presentation of results. | Methods: Data extraction and analysis |
| Synthesis methods | 13a | Describe the processes used to decide which studies were eligible for each synthesis (e.g. tabulating the study intervention characteristics and comparing against the planned groups for each synthesis (item #5)). | Methods: Study selection |
|  | 13b | Describe any methods required to prepare the data for presentation or synthesis, such as handling of missing summary statistics, or data conversions. | Methods: Data extraction and analysis |
|  | 13c | Describe any methods used to tabulate or visually display results of individual studies and syntheses. | Methods: Data extraction and analysis |
|  | 13d | Describe any methods used to synthesize results and provide a rationale for the choice(s). If meta-analysis was performed, describe the model(s), method(s) to identify the presence and extent of statistical heterogeneity, and software package(s) used. | Methods: Data extraction and analysis |
|  | 13e | Describe any methods used to explore possible causes of heterogeneity among study results (e.g. subgroup analysis, meta-regression). | Methods: Data extraction and analysis |
|  | 13f | Describe any sensitivity analyses conducted to assess robustness of the synthesized results. | Methods: Data extraction and analysis |
| Reporting bias assessment | 14 | Describe any methods used to assess risk of bias due to missing results in a synthesis (arising from reporting biases). | Methods: Data extraction and analysis |
| Certainty assessment | 15 | Describe any methods used to assess certainty (or confidence) in the body of evidence for an outcome. | Methods: Data extraction and analysis |
| RESULTS | | |  |
| Study selection | 16a | Describe the results of the search and selection process, from the number of records identified in the search to the number of studies included in the review, ideally using a flow diagram. | Results, Fig1 |
|  | 16b | Cite studies that might appear to meet the inclusion criteria, but which were excluded, and explain why they were excluded. | Results, Table1 |
| Study characteristics | 17 | Cite each included study and present its characteristics. | Results, Table1 |
| Risk of bias in studies | 18 | Present assessments of risk of bias for each included study. | Results: Quality and risk of bias within studies, Supplementary Table1 |
| Results of individual studies | 19 | For all outcomes, present, for each study: (a) summary statistics for each group (where appropriate) and (b) an effect estimate and its precision (e.g. confidence/credible interval), ideally using structured tables or plots. | Results: Meta-analysis, Sensitivity analysis, Table2-3, Fig2-3 |
| Results of syntheses | 20a | For each synthesis, briefly summarise the characteristics and risk of bias among contributing studies. | Results: Meta-analysis, Sensitivity analysis, Table2-3, Fig2-3 |
|  | 20b | Present results of all statistical syntheses conducted. If meta-analysis was done, present for each the summary estimate and its precision (e.g. confidence/credible interval) and measures of statistical heterogeneity. If comparing groups, describe the direction of the effect. | Results: Meta-analysis, Sensitivity analysis, Table2-3, Fig2-3 |
|  | 20c | Present results of all investigations of possible causes of heterogeneity among study results. | Results: Meta-analysis, Sensitivity analysis, Table2-3, Fig2-3 |
|  | 20d | Present results of all sensitivity analyses conducted to assess the robustness of the synthesized results. | Results: Sensitivity analysis, Supplementary Table2-3, Supplementary Table 4-5 |
| Reporting biases | 21 | Present assessments of risk of bias due to missing results (arising from reporting biases) for each synthesis assessed. | Results: Publication bias, Supplementary Fig4 |
| Certainty of evidence | 22 | Present assessments of certainty (or confidence) in the body of evidence for each outcome assessed. | Results: Sensitivity analysis, Supplementary Table2-3, Supplementary Table 4-5 |
| DISCUSSION | | |  |
| Discussion | 23a | Provide a general interpretation of the results in the context of other evidence. | Discussion |
|  | 23b | Discuss any limitations of the evidence included in the review. | Discussion: Limitations |
|  | 23c | Discuss any limitations of the review processes used. | Discussion: Limitations |
|  | 23d | Discuss implications of the results for practice, policy, and future research. | Discussion: Conclusions |
| OTHER INFORMATION | | |  |
| Registration and protocol | 24a | Provide registration information for the review, including register name and registration number, or state that the review was not registered. | Methods: Protocol |
|  | 24b | Indicate where the review protocol can be accessed, or state that a protocol was not prepared. | Methods: Protocol |
|  | 24c | Describe and explain any amendments to information provided at registration or in the protocol. | NA |
| Support | 25 | Describe sources of financial or non-financial support for the review, and the role of the funders or sponsors in the review. | Additional information |
| Competing interests | 26 | Declare any competing interests of review authors. | Additional information |
| Availability of data, code and other materials | 27 | Report which of the following are publicly available and where they can be found: template data collection forms; data extracted from included studies; data used for all analyses; analytic code; any other materials used in the review. | Additional information |
